# Response of extreme haloarchaeon *Haloarcula argentinensis* RR10 to simulated microgravity in clinorotation

**DOI:** 10.1007/s13205-016-0596-2

**Published:** 2017-04-11

**Authors:** Rebecca Thombre, Vinaya Shinde, Jyotsana Dixit, Sagar Jagtap, Pandit B. Vidyasagar

**Affiliations:** 1 Department of Biotechnology, Modern College of Arts, Science and Commerce, Shivajinagar, Pune, Maharashtra 411005 India; 2 School of Basic Medical Sciences, Savitribai Phule Pune University, Ganeshkhind, Pune, Maharashtra 411007 India; 3 Department of Physics, H. V. Desai College, Pune, Maharashtra 411002 India; 4 Swami Ramanand Teerth Marathwada University, Nanded, Maharashtra 431606 India

**Keywords:** Simulated microgravity, Haloarchaea, *Haloarcula argentinensis*, ‘Salt-in’ strategy, Antimicrobial resistance, Physiological mechanisms, Growth kinetics

## Abstract

Gravity is the fundamental force that may have operated during the evolution of life on Earth. It is thus important to understand as to what the effects of gravity are on cellular life. The studies related to effect of microgravity on cells may provide greater insights in understanding of how the physical force of gravity shaped life on Earth. The present study focuses on a unique group of organisms called the Haloarchaea, which are known for their extreme resistance to survive in stress-induced environments. The aim of the present investigation was to study the effect of simulated microgravity on physiological response of extremely halophilic archaeon, *Haloarcula argentinensis* RR10, under slow clinorotation. The growth kinetics of the archaeon in microgravity was studied using the Baryani model and the viable and apoptotic cells were assessed using propidium iodide fluorescent microscopic studies. The physiological mechanism of adaptation was activation of ‘salt-in’ strategy by intracellular sequestration of sodium ions as detected by EDAX. The organism upregulated the production of ribosomal proteins in simulated microgravity as evidenced by Matrix-assisted laser desorption ionization Time of flight–Mass Spectrophotometry. Simulated microgravity altered the antibiotic susceptibility of the haloarchaeon and it developed resistance to Augmentin, Norfloxacin, Tobramycin and Cefoperazone, rendering it a multidrug resistant strain. The presence of antibiotic efflux pump was detected in the haloarchaeon and it also enhanced production of protective carotenoid pigment in simulated microgravity. The present study is presumably the first report of physiological response of *H. argentinensis* RR10 in microgravity simulated under slow clinorotation.

## Introduction

Outer space and planets, excluding Earth, has an environment that is characterized by harsh inhabitable conditions. The profound disparity in gravity, radiation, pressure, atmosphere, oxygen, salinity and nutrients are responsible for the hostile and extreme conditions pernicious for growth of microbial life in outer space. One of the contrasting differences in the environment of Earth as compared to the environment existing in spacecraft, spaceflights and international space stations located in outer space is dissimilitude in gravitational forces. The gravity in these space locations is diminished from normal gravity (NG) of 1*g* (gravitational force of Earth) to 10^−2^ to 10^−6^ *g* called as microgravity. Microgravity is responsible for the experience of weightlessness in spaceflights and spacecrafts and recently studies related to the effect of microgravity on cells have garnered profound significance. Effect of microgravity can be studied in international space stations or the microgravity conditions can be simulated in a laboratory. The essential change in environment observed in spaceflights and spacecraft is the change in the gravitational force. In spaceflights and international space stations, the gravitational force is diminished to such an extent that objects appear weightless as a consequence of which astronauts and objects float inside the spacecraft. Though the gravitational force doesn’t become zero, it is reduced to small amount (10^−6^ *g*) and thus is called as microgravity.

Study of microgravity in the laboratory can be performed by simulation of microgravity in specialized bioreactors that have low shear dynamics and such an environment is called as low-shear modeled microgravity (LSMMG) environment. Specialized rotating ground based bioreactors similar to suspension culture vessels used in tissue culture are utilized for generation of LSMMG and these are known as high-aspect-ratio vessel (HARV) (Purevdorj-Gage et al. 2006). A similar device called as a Rotary Cell Culture System manufactured by Synthecon (Houston, TX) has been used to simulate microgravity (Xu et al. 2015). The effect of microgravity on cells can also be studied using a clinostat that rotates at a low speed along a horizontal axis so as to negate the forces of gravity and simulate a microgravity environment (Jagtap et al. 2011).

A clinostat is a device that can be used to simulate microgravity conditions that exist in outer space or spaceflights for microbial suspension cell cultures (Klaus et al. 1997). The simulated microgravity is created by rotation of a cylinder at constant velocity completely filled with a liquid broth medium containing cells devoid of any air bubbles or spaces (Klaus et al. 1998).

These studies have been carried out in the clinostat at slow rotation (Jagtap et al. 2011) (ranging from 0.25 to 3 rpm) and at higher rotations (Luna et al. 2015) (30–75 rpm). When the clinostat is operated at proper rotation, the cells neither sediment nor adhere to the container wall thereby reducing the relative fluid motion and simulating weightlessness as experienced in spaceflights. The term “simulated microgravity” can thus be defined as a state resultant of clinorotation, in which the force due to gravity (*g*) is not completely obliterated rather the rotation at a constant velocity results in the *g*-vector being time-averaged to near-zero (Klaus et al. 1997, 1998). The response of haloarchaea *Haloferax mediterranei* DSM 1411^T^ and *Halococcus dombrowskii* DSM 14522^T^to microgravity simulated in RCCS, Synthecon has been previously investigated (Dornmayr-Pfaffenhuemer et al. 2011). However, there are no studies related to the response of haloarchaea in clinorotation.

In the present study, a clinostat under slow rotation was used for studying the effect of simulated microgravity (SMG) on halophilic archaea. Halophiles are considered as model organisms for stress related experiments and astrobiology related investigations (DasSarma 2006). Haloarchaea are halophilic archaea that have a mandatory requirement of 1.5 mol l^−1^ NaCl for growth and extreme haloarchaea can survive up to 5.1 mol l^−1^NaCl (Thombre et al. 2016a). They have the inherent and acquired ability to survive and adjust in manifold stresses which is the key factor for studies related to astrobiology. Haloarchaea have also been known to survive in halite crystals for long periods and survival ability was of paramount importance in application of survival studies of haloarchaea in outer space environments like the European Space Agency’s, Biopan facility and International Space Station (Mancinelli et al. 1998; Stan-Lotter et al. 2002; Fendrihan et al. 2006). The studies related to survival of haloarchaea in microgravity are crucial in contributing valuable information regarding exploration of life in outer space. Besides these organisms, spores and coccoid cells are considered as ‘micronauts’ that are microorganisms that can survive travel from one planet to another (Dornmayr-Pfaffenhuemer et al. 2011). The survival mechanisms of haloarchaea during stress-conditions may provide useful insights to the possibility of their survival elsewhere.

The present study focuses on studying the growth kinetics of extremely halophilic archaeon, *Haloarcula argentinensis* RR10 in simulated microgravity and assessing the adaptation response of the organisms under clinorotation. The haloarchaeon utilizes a combination of physiological responses including intracellular sequestration of sodium ions, production of stress proteins and counteraction of cellular damage due to oxidative stress balanced by production of pigment to adapt to simulated microgravity. The response and plausible mechanism of survival and adaptation of haloarchaea during simulated microgravity is being reported presumably for the first time in *H. argentinensis* RR10.

## Materials and methods

### Growth of microorganism

The organism used in the present study was haloarchaeon, *H. argentinensis* strain RR10 (GenBank/EMBL/DDBJ accession number KP712898, MCC 2923) isolated in our laboratory previously from salterns of Mumbai, India (Thombre et al. 2016b). It is an extreme halophile that requires 3–5 mol l ^−1^sodium chloride for growth. It was cultured in broth culture in an orbital shaker at 40 °C at 100 rev min ^−1^ in Sehgal and Gibbons (SG) medium containing (g/L) casamino acids (7.5), yeast extract (10), potassium chloride (2), trisodium citrate (3), magnesium sulfate (20) and pH- 7.5 supplemented with 4.28 mol l^−1^ sodium chloride (Sehgal and Gibbons 1960).

### Growth kinetic studies of *Haloarcula argentinensis* RR10 during simulated microgravity generated in clinostat

The growth kinetics of *H. argentinensis* RR10 under simulated microgravity (SMG) and normal gravity (NG) was studied by exposing the organism to SMG in a clinostat. The microgravity conditions were simulated in a 1-D clinostat developed in-house in the Department of Physics, S.P. Pune University, India (Jagtap et al. 2011). Exponential phase culture of *H. argentinensis* RR10 (~10^6^ CFU/ml) in SG medium was dispensed in 50 ml sterile cylindrical syringes and rotated in horizontal axis generating simulated microgravity of 8.94 × 10^−5^×*g* at 40 °C. The control consisted of cells of *H. argentinensis* RR10 in 50 ml sterile syringe maintained in normal gravity (NG) conditions (1×*g*) at 40 °C without agitation. Samples were withdrawn aseptically every 24 h from the medium maintained in SMG and NG conditions and the growth was measured in terms of absorbance at 600 nm using UV Visible Spectrophotometer (UV-1800, Shimadzu, Japan). The growth kinetics were studied using the Baranyi model (Baranyi and Roberts 1994) and the curve-fitting DMFit programme and the growth rate (*μ*), generation time (*g*) and the specific growth rate constant (*k*) and *λ* (lag phase) were calculated from the growth curve as described earlier (Salgaonkar et al. 2016; Metris et al. 2006; Robinson et al. 2005; Thombre et al. 2016c).

### Assessment of viability of haloarchaea in SMG using propidium iodide fluorescent staining

The effect of SMG on viability of haloarchaea was studied using propidium iodide staining and visualization using fluorescence microscopy. Propidium iodide stains the cells that exhibit damaged membrane red in color indicating non-viable, metabolically injured or necrotic cells. Propidium iodide cannot penetrate intact membranes of live cells, hence viable cells don’t take up the red color (Stan-Lotter et al. 2002). Briefly, the cells exposed to SMG and NG were stained with propidium iodide solution (Sigma, Germany) and observed under Fluorescent microscope (AV 10-Zeiss, Germany) with Apotome and 400 X filter using the Axiovision software for imaging and the number of damaged cells (cells stained red with propidium iodide) were counted (Rieger et al. 2011). The percentage of viable cells was calculated as:  % viability = (number of viable cells counted/total number of cells counted) × 100.

### Study of ‘salt-in strategy’ of osmoadaptation in haloarchaea during SMG by energy dispersive X-ray spectroscopy (EDAX) analysis

On exposure to simulated microgravity, haloarchaea accumulate intracellular ions as a part of the ‘salt-in strategy’ of adaptation. The archaeal intracellular ion accumulation in response to SMG was studied using EDAX analysis (Jeol, Japan). The sample preparation of *H. argentinensis* RR10 cells exposed to simulated microgravity and normal gravity for EDAX was as detailed previously (Thombre et al. 2016b).

### Production of protective carotenoid pigments produced in response to SMG

The production of protective red carotenoid pigment produced by *H. argentinensis* RR10 in response to SMG and NG was studied. The total pigment content present was estimated by measuring the absorbance of the culture broth at 490 nm (*λ*_max_ of haloarchaeal carotenoid) using UV–Vis Spectrophotometer (UV-1800, Shimadzu, Japan) (Abbes et al. 2013).

### Effect of SMG on antibiotic resistance of *H. argentinensis* RR10 and detection of antibiotic efflux pumps

The changes in the antibiotic resistance pattern of *H. argentinensis* RR10 in response to SMG and NG were studied by disc diffusion method as per Clinical and Laboratory Standards Institute (CLSI) guidelines (2012) and Shinde and Thombre (2016). Briefly, 100 µl of the culture (absorbance corresponding to 0.5 Mc Farland standard) exposed to SMG and NG (7th day) was spread on SG medium and antibiotic discs of Augmentin, Norfloxacin, Nalidixic acid, Imipenem, Tobramycin, Cefoxitin, Cefoperazone and Piperacillin/Tazobactam (HiMedia, India) were placed aseptically on it and incubated for 7–10 days at 40 °C. The results were interpreted as resistant or sensitive using interpretive criteria according to the CLSI guidelines (CLSI 2014). Detection of antibiotic resistance efflux pumps responsible for antibiotic resistance was performed by slightly modified ethidium bromide-agar cartwheel method (Martins et al. 2011; Shinde and Thombre 2016). Cells of *H. argentinensis* RR10 (absorbance adjusted to 0.5 of Mc Farland standard) exposed to SMG and NG were streaked on SG agar supplemented with 4.8 mol l^−1^ sodium chloride containing EtBr (0–2.5 mg L^−1^) divided into radial sectors and incubated at 40 °C for 5–7 days till growth appeared. The plates were examined under UV trans illuminator and the minimum concentration of EtBr (MIC_EtBr_) that produced fluorescence was recorded.

### SDS-PAGE of differential proteins expressed in haloarchaea during SMG

After exposure to simulated microgravity, the proteins expressed in cytosol of *H. argentinensis* RR10 were subjected to separation using 1-D SDS PAGE as described previously (Thombre et al. 2016b; Otte et al. 2014). The protein bands obtained after staining from the cells exposed to SMG and NG were compared and the differentially expressed protein bands in SMG sample were identified and selected for peptide mass finger printing. The selected bands were excised from the gel and trypsinized as described by Otte et al. (2014). Briefly, the protein sample was trypsinized with trypsin digestion buffer (50 mM NH_4_HCO_3_, 5 mM CaCl_2_, 12.5 ng/µl trypsin) and the peptide digest extract was prepared as described earlier (Thombre et al. 2016b).

### Identification of differential proteins using MALDI TOF-MS and MASCOT analysis

The peptide digest extracted from the gel piece (1 µl) was premixed with equal volume of matrix and spotted on a matrix-assisted laser desorption ionization (MALDI) plate as per manufacturer’s instructions. The Peptide mass fingerprint (PMF) was obtained using MALDI TOF-mass spectrometer (Ultraflex II, Bruker Daltonics, Germany) in the reflector mode. The data generated was analyzed using Swiss-Prot database using MASCOT search engine(Matrix Science, London, United Kingdom) with a peptide mass tolerance of 100 ppm (Trotter et al. 2015; Govekar et al. 2012; Thombre et al. 2016b).

### Statistical Analysis

All experiments were performed in triplicates. Statistical analysis was performed using Excel 2016. All values were expressed as the mean ± standard error (SE). Statistical significance between groups was calculated using the paired Student’s *t* test and the value of *p* < 0.10 was considered to be statistically significant.

## Results

### Growth kinetic studies of *Haloarchaea* in simulated microgravity generated in clinostat

**Fig. 1:**
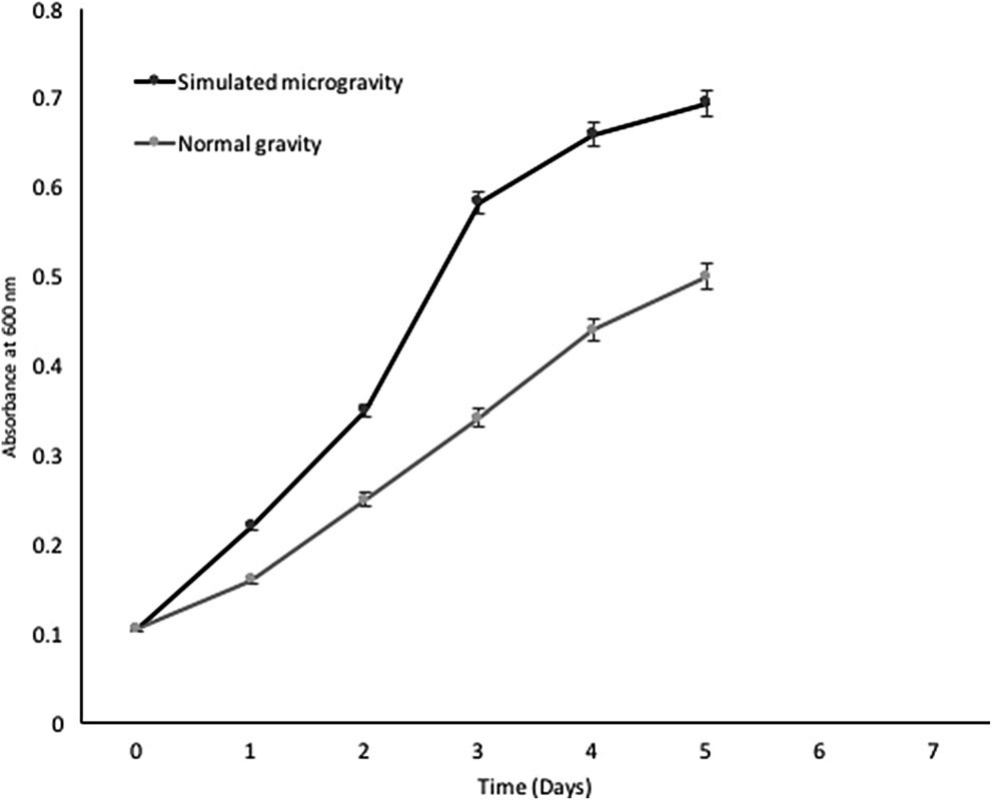
Effect of simulated microgravity and normal gravity on growth of *Haloarcula argentinensis* RR10

**Fig. 2:**
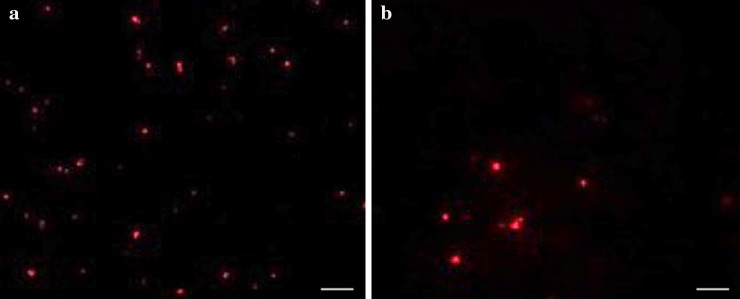
**a** Fluorescence images of *Haloarcula argentinensis* RR10 exposed to normal gravity stained by propidium iodide. **b** Fluorescence images of *Haloarcula argentinensis* RR10 exposed to simulated microgravity stained by propidium iodide

**Fig. 3:**
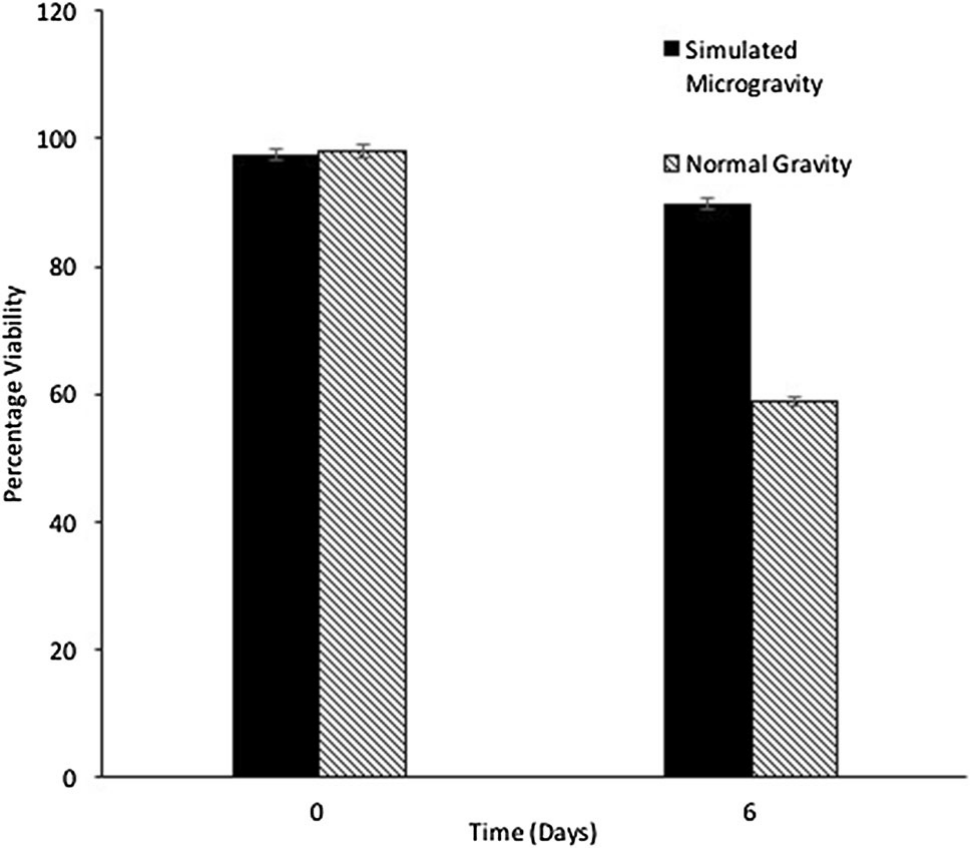
Percentage viability of haloarchaeal cells exposed to simulated microgravity and normal gravity detected by propidium iodide staining

The growth kinetics of *H.* argentinensis RR10 in simulated microgravity under clinorotation was studied. Under the effect of simulated microgravity, the growth was faster as compared to the same culture incubated under normal gravity conditions (Fig. 1). The difference in growth between normal and simulated microgravity was statistically significant (*t* value = 1.4028 and *p* value = 0.095475 at *p* < 0.10) and growth was higher in simulated microgravity. The growth kinetics in simulated microgravity and normal gravity were studied using the growth curve plots and no lag phase (*λ*) was observed in both conditions. The doubling time or generation time (*t*) was 8 and 20 h and growth rate constant (*k*) was 0.0866 and 0.03465 h^−1^ for simulated and normal gravity respectively. The Monod’s maximum specific growth rate was also calculated using the Baryani model and DMFit curve-fitting programme and the growth rate (*µ*) was calculated as 0.12 and 0.08 h^−1^ for simulated microgravity and normal gravity, respectively. It is apparent from the growth kinetic studies that the haloarchaeon demonstrates maximum growth in simulated microgravity. The viability of the cells exposed to altered gravity was assessed using fluorescent microscopy using propidium iodide. Propidium iodide is impermeable to viable cells that maintain cell membrane integrity. However, due to stress, cells undergo necrosis and exhibit damaged cell membrane due to loss of membrane integrity as a result of which the propidium iodide is taken up by the cells and it demonstrates red fluorescence in non viable cells. When haloarchaea were exposed to simulated microgravity, they demonstrated faster growth and more number of viable cells as compared to haloarchaea cultured under normal gravity (Fig. 2). The number of non viable cells demonstrating red fluorescence was significantly higher under normal gravity in comparison to the cells exposed to SMG (Fig. 3). Besides, it was observed that cellular aggregation under SMG was higher and clumping of cells was observed in simulated microgravity conditions as seen in Fig. 2b. However, under normal gravity conditions, lesser aggregation was seen as evidenced by the smaller size of cells in NG (Fig. 2a). It is suggested that further studies using commercially available Live-Dead staining kits and FACS could provide deeper insights about viability of cells and the presence of dead, damaged and viable but unculturable cells in simulated microgravity and normal gravity.

### Study of ‘salt-in strategy’ of osmoadaptation in haloarchaea during SMG by energy dispersive X-ray spectroscopy (EDAX) analysis

**Fig. 4:**
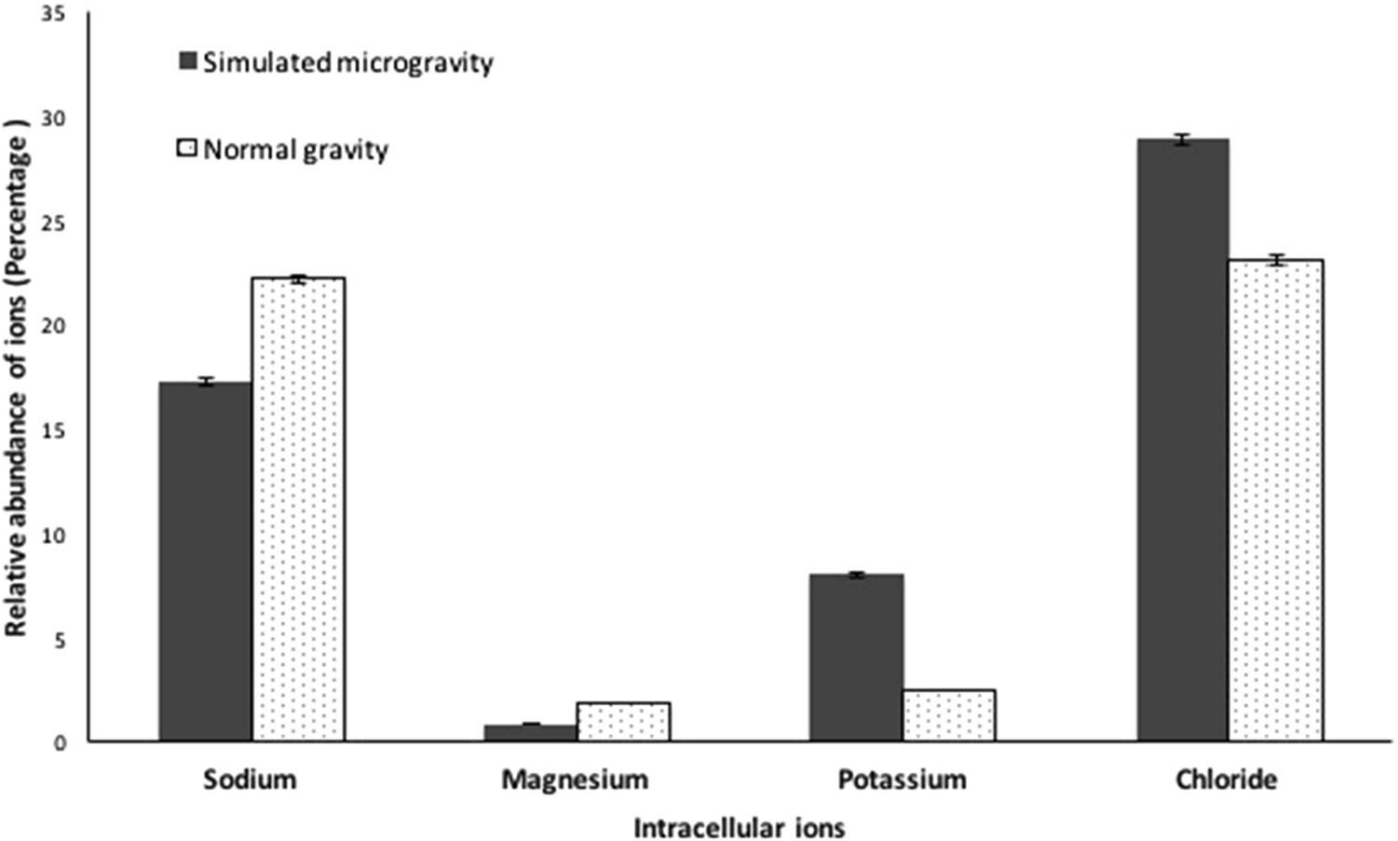
Relative percentage of intracellular archaeal ion accumulation by *Haloarcula argentinensis* RR10 when exposed to simulated microgravity and normal gravity detected by EDAX

On exposure to harsh conditions, haloarchaea utilize the ‘salt-in strategy’ to counteract the effect of osmotic stress. Using this strategy, the organism sequesters salt intracellularly to counter balance the gradients of salts created during stress. In order to study if this strategy is utilized by *H. argentinensis* RR10 during simulated microgravity, a detection of the intracellular archaeal ions sequestered in simulated microgravity and normal gravity was performed. It was observed that in simulated microgravity, the haloarchaeon increased the sequestration sodium, potassium and chloride ions significantly (Fig. 4). Maximum sequestration during simulated microgravity was of sodium ions and the trend in ion accumulation in SMG was Na > Cl > K and for NG is Cl > Mg > Na > K.

### Production of protective carotenoid pigments produced in response to simulated microgravity

**Fig. 5:**
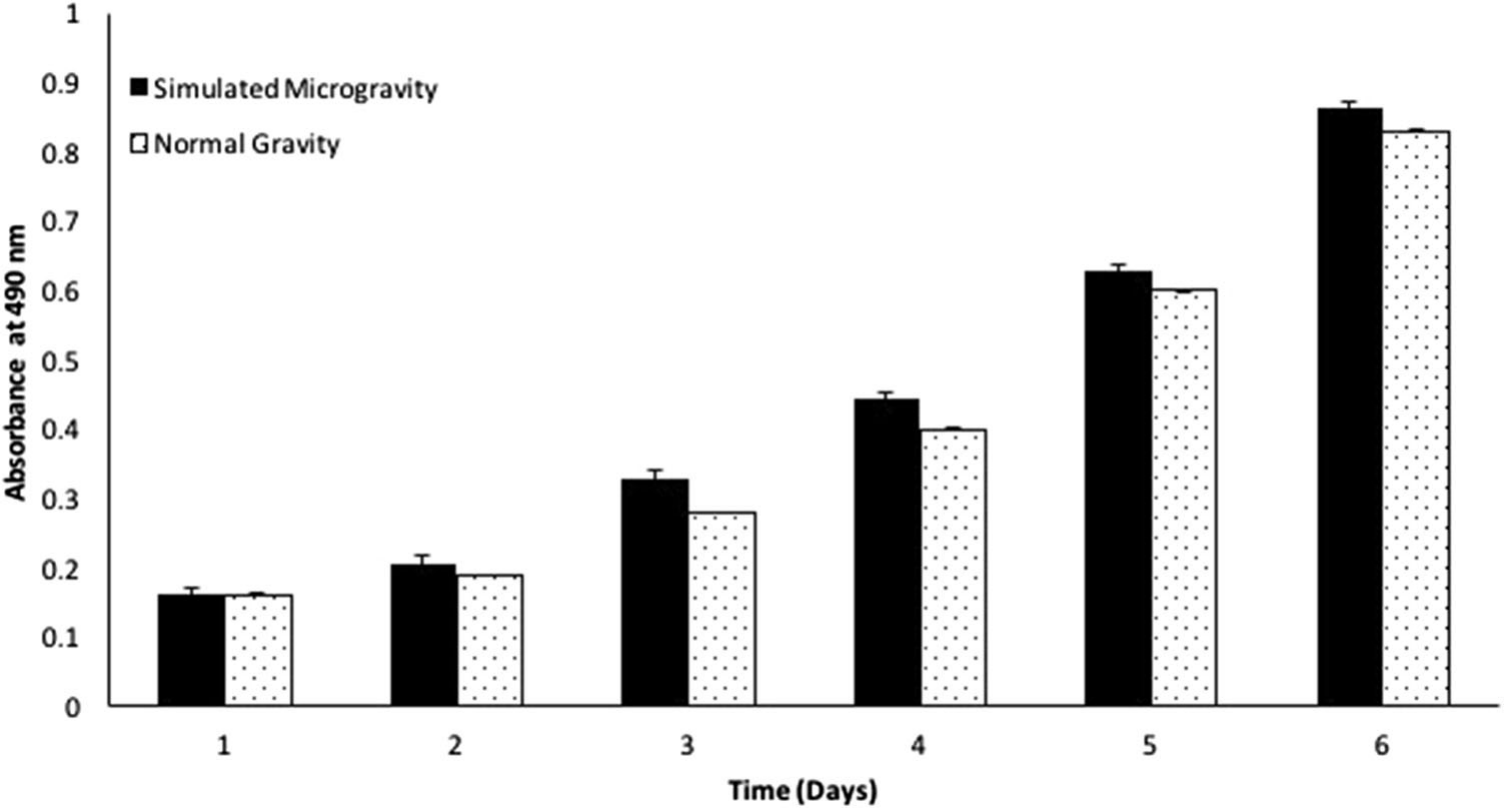
Effect of simulated microgravity and normal gravity on total carotenoid pigment production by *Haloarcula argentinensis* RR10

Red-orange colored pigments are produced by haloarchaea to tackle oxidative stress caused by external environmental factors affecting the culture during growth. It was observed that when *H. argentinensis* RR10 was exposed to SMG, it produced significantly higher amount of deep red–orange pigmentation in comparison to its pigment production in normal gravity (Fig. 5). The UV–Vis spectral scan and absorption maxima of the pigment indicates that the carotenoid produced resembles the pigment bacterioruberin (Abbes et al. 2013).

### Effect of SMG on antibiotic resistance of *H. argentinensis* RR10 and detection of antibiotic efflux pumps

The wild type strain of *H. argentinensis* RR10 is sensitive to the antibiotics Augmentin, Norfloxacin, Imipenem, Tobramycin, Cefoxitin, Cefoperazone and Piperacillin/Tazobactam and resistant to Nalidixic acid under normal gravity conditions. On exposure to simulated microgravity, *H. argentinensis* RR10 developed resistance to many antibiotics as depicted in Table 1. As the isolate demonstrated change in its antibiotic resistance after exposure to SMG, the possible involvement of antibiotic efflux pump was studied by modified ethidium bromide cartwheel assay. It was observed that cells of *H. argentinensis* RR10 showed the upregulation of efflux pump activity in SMG conditions as evidenced by visualization of fluorescence under UV-trans illuminator. The minimum concentration of EtBr (MIC_EtBr_) that produced fluorescence of the haloarchaeal culture when exposed to normal gravity was 2 mg L^−1^. However, on exposure to simulated microgravity, the efflux pump activity was seen in the cartwheel assay at a much lower concentration of MIC_EtBr_ = 1.5 mg L^−1^. There was increase in the antibiotic resistance of the archaeon on exposure to simulated microgravity and the organism responded by over expressing the efflux pumps. The fluorescence produced by the cells when exposed to simulated microgravity was greater as compared to the cells cultured in normal gravity as observed by the ethidium bromide cartwheel assay.

**Table 1:** The antibiotic resistance profile of *Haloarcula argentinensis* RR10 exposed to Simulated microgravity and normal gravity conditions

S. no.	Antibiotic	Concentration (µg)	Class/type of antibiotic	Interpretive criteria for zone diameter^a^ (mm)			Antibiotic resistance profile of *Haloarcula argentinensis* RR10	
				S	I	R	Normal gravity	Simulated microgravity
1	Augmentin	30	Penicillin + clavulanic acid	–	–	–	S	R
2	Norfloxacin	10	Fluoroquinolone	≥17	13–16	12≤	S	R
3	Nalidixic acid	30	Quinolone	≥19	14–18	13≤	R	R
4	Imipenem	10	Carbapenem	≥23	20–22	19≤	S	S
5	Tobramycin	10	Aminoglycoside	≥15	13–14	12≤	S	R
6	Cefoperazone	75	Cephalosporin	≥21	16–20	15≤	S	R
7	Cefoxitin	30	Cephamycin	≥18	15–17	14≤	S	S
8	Piperacillin/tazobactam	100/10	β-lactam + β-lactamase inhibitor	≥21	18–20	17≤	S	S

*R* resistant, *I* intermediate, *S* sensitive, *nd* not determined

^a^Interpretive criteria of Gram-negative bacteria as per guidelines of CLSI M100-S24 for disk diffusion

### Differential proteins expressed in response to SMG in *H. argentinensis* RR10

We examined the effects of simulated gravity conditions on *H. argentinensis* at protein level using a non targeted proteomic 1D-DIGE approach and MALDI TOF analysis. Five differential bands were identified using MS followed by MASCOT analysis (Table 2). Majority of the peptides identified in response to simulated microgravity were ribosomal proteins ranging in the size 10–17 kDa. The abundance of ribosomal proteins unregulated was a significant finding in terms of response to simulated microgravity.

**Table 2:** Differential proteins expressed by *Haloarcula argentinensis* RR10 in SMG studied using peptide mass fingerprinting (PMF)

S. no.	Description of putative peptide/protein	Gene	Putative pI	Putative molecular weight (Da)
1	50S ribosomal protein L22	rpl22	5.0	17,191.480
2	30S ribosomal protein S8	rps8	7.7	14,305.200
3	Translation initiation factor 2 subunit beta	eif2b	8.9	16,215.180
4	30S ribosomal protein S11	rps11	11.6	14,082.540
5	50S ribosomal protein L31e	rpl31e	4.8	10,129.080

## Discussion

Understanding the evolution of life-forms on Earth and their survival during initial extremely unfavorable environmental conditions is of paramount importance. With the progression of life, the inherent and acquired abilities to survive in multiple stresses especially altered gravity may have been a key factor for survival of organisms. Haloarchaea are a unique group of poly-extremophilic organisms with unusual features and are known to survive multiple stresses. Their physiological robustness and ability to survive in the presence of steep fluctuations in temperature, atmosphere, oxygen, radiations, salinity, perchlorate, epsomite, gravity and numerous other stresses has always intrigued scientists. They are apt model exo-philes to study possible effect and adaptability of life forms in spacecraft, in altered gravity like microgravity and hypergravity, as well as in outer space (Thombre et al. 2017). Currently, studies aim at understanding the effect of changes in mechanical forces that occur in microgravity and other low-shear environments on different microbial parameters (Nickerson et al. 2004). Microgravity is known to affect the physiology of cells, gene expression and increase the antibiotic resistance of microorganisms (Xu et al. 2015). Previously studies have been conducted on the effect of space like conditions including microgravity on growth of *Escherichia coli* (Xu et al. 2015), *Salmonella typhimurium* (Wilson et al. 2007), *Saccharomyces cerevisiae* (Purevdorj-Gage et al. 2006) and plants (Jagtap et al. 2011). The effect of simulated microgravity in clinorotation have been studied using *E. coli* (Brown et al. 2002; Arunasri et al. 2013) and the fungi *Aspergillus niger* and *Candida albicans* (Yamazaki et al. 2012). However, this is the first report on effect of simulated microgravity on extremely halophilic archaeon *H. argentinensis* RR10 in clinorotation. This strain was isolated from a harsh hypersaline thalossohaline salterns of Mumbai, India. This haloarchaeon was selected for the present study as it is a poly-extremophile and it demonstrated survival over a wide range of salinity, magnesium concentration, temperature and pH.

On exposure to simulated microgravity, the haloarchaeon *H. argentinensis* RR10 still continued to demonstrate faster growth as evidenced by increase in absorbance, lesser doubling time and higher percentage viability observed by fluorescence microscopy in comparison to the cells cultured in normal gravity. *E. coli* when exposed to simulated microgravity showed a similar statistically significant increase in growth as compared to *E. coli* incubated in NG (Xu et al. 2015). The exact responses and adaptations that haloarchaea elicit in stresses and such altered gravity environment is still unidentified. The general strategies these organisms utilize in ionic stresses are the ‘salt-in strategy’ and the ‘organic osmolyte strategy’ also referred to as compatible solute strategy. In the organic osmolyte strategy, haloarchaea synthesize or carry out the uptake of organic compatible solutes in the cytosol. Thus, the osmotic potential is counter balanced despite maintenance of low salt intracellularly. The compatible solutes are ectoine, glycine betaine, trehalose, sorbitol, glycerol and amino acid derivatives. This strategy is more common in halophilic bacteria. The ‘salt-in strategy’ is employed by true halophiles, including halophilic archaea and extreme haloarchaea (Shivanand and Mugeraya 2011). In this mechanism the organism selectively sequesters the cations (sodium or potassium) inside the cytoplasm instead of synthesizing organic osmolytes so as to maintain the ionic concentration in the cell equivalent or higher than the external environment (Jensen et al. 2015). As the ‘salt- in strategy’ is common in extreme haloarchaea, occurrence of this strategy in simulated microgravity was studied by detecting the intracellular archaeal accumulation of ions by *H. argentinensis* RR10 using EDAX analysis. In the current study, *H. argentinensis* RR10 demonstrated greater sequestration of chloride followed by sodium ions in NG as compared to SMG conditions. Presence of a light independent Cl^−^ transport channel (Nad and Nac complex) has been identified in the genome of *Haloarcula marismortui*. Similarly, haloarchaea possess halorhodopsin which is a light gated ion channel or a light driven chloride pump that is causal for chloride accumulation inside the cell (Jensen et al. 2015). It is known that the archaeon *H. argentinensis* has presence of retinal proteins in the membrane (Ihara et al. 1997). The isolate may thus use halorhodopsin or the light independent Na^+^/Cl^−^ symporter or transporter to sequester chloride ions in cytoplasm during its growth in normal gravity of 1*g*. The chloride anions may possibly react with the hydroxyl radicals in the cell to generate lesser reactive chloride radicals that tend to mitigate the deleterious effects of oxidative damage caused by more reactive hydroxyl radicals. Generally, haloarchaea increase the intracellular influx of potassium ions instead of sodium ions, driven by proton motive force via a Trk K^+^/H^+^ symport while growth in osmotic stress (Jensen et al. 2015). However, in *H. argentinensis* RR10, the sequestration of sodium ions is higher as compared to potassium ions. This may be due to the plausible preferential sequestration of sodium ions and replacement of potassium ion when exposed to SMG.

The study of differential proteins expression was studied in *H. argentinensis*. The differences in the bands present in the protein fraction of the isolate incubated in SMG and NG was apparent. Previous studies have reported a notable difference in protein profile when *H. mediterranei* DSM 1411^T^ and *Halococcus dombrowskii* DSM 14522^T^ were exposed to SMG in exponential phase (Dornmayr-Pfaffenhuemer et al. 2011). However, the proteins expressed in haloarchaea in response to simulated microgravity were not identified previously (Dornmayr-Pfaffenhuemer et al. 2011). Moreover, when the proteins are separated using 2-D gel electrophoresis, the majority of haloarchaeal proteins being acidic, aggregate and cluster as they are in a narrow pI range (4.2–5.2) causing difficulty in separation and study of these proteins (Thombre et al. 2016b). To avoid these problems in the present study, we directly excised the differential protein band expressed in simulated microgravity conditions in 1-D SDS-PAGE gels and trypsinized them and attempted to identify the peptide mass fingerprint generated using MALDI-TOF MS (Trotter et al. 2015). The major peptide samples analyzed and identified by MASCOT revealed the presence of 50S ribosomal protein L22, 50S ribosomal protein L31e, 30S ribosomal protein S8, 30S ribosomal protein S11and Translation initiation factor 2 subunit-betain *H. argentinensis*. Studies related to the proteome analysis of cells exposed to SMG are limited. The proteomic analysis of *Daphnia* exposed to SMG demonstrated upregulation of proteins related to actin microfilament organization, protein folding and energy metabolism-related proteins (Trotter et al. 2015). The genomic studies related to haloarchaea reveal that genes encoding for functional classes of chaperones (CHP), signal transduction (SIG) and cellular processes (CP) are up regulated during exponential phase as compared to stationary phase (Lange et al. 2007). The changes in environmental conditions leads to stress which is the cardinal causal factor responsible for upregulation of chaperonins, proteasomal proteins and small heat shock proteins (sHSP’s) in haloarchaea (Thombre and Oke 2015; Thombre et al. 2016b). The genomic data of *E. coli* K12 MG1655(cultured in microgravity under clinorotation) analyzed based on the DAVID geneontology (GO) term enrichments revealed that genes for DNA transcription, helicases, multidrug efflux system and small RNA were upregulated and genes involving stress proteins were downregulated (Arunasri et al. 2013). A global response to simulated microgravity involving a general regulator of transcription has been envisaged in bacteria (Wilson et al. 2007).In the present study, the maximum proteins differentially expressed were ribosomal proteins indicating activation transcription process. This indicates that microgravity does not hamper growth of the organisms, on the contrary the archaeon continues to demonstrate active transcription process.

Haloarchaea are extremely resistant to antibiotics and when cells are exposed to microgravity, their antibiotic resistance increases markedly (Dornmayr-Pfaffenhuemer et al. 2011). When *H. argentinensis* was exposed to SMG, it altered the resistance pattern of the organism and it developed resistance to many antibiotics like Augmentin, Norfloxacin, Tobramycin and Cefoperazone in addition to its resistance to Nalidixic acid. The antibiotic susceptibilities of the organism are altered during microgravity and analogous findings of increase in susceptibility to antibiotic flucanozole by *C. albicans* in response to SMG stress has been reported (Jiang et al. 2014). Currently the data regarding drug resistance and break points as per Clinical Laboratory Standards Institute (CLSI) and European Committee on Antimicrobial Susceptibility Testing (EUCAST) for classification of multi drug resistance in haloarchaea is not available. As per CLSI and EUCAST an organism is considered multi drug resistant if it develops resistance to at least one agent in three or more antimicrobial categories (Mapara et al. 2015; Shinde and Thombre 2016). Since *H. argentinensis* RR10 developed resistance to at least one agent in three or more antimicrobial categories (viz. Aminoglycoside, Quinolone, Fluoroquinolone, Cephalosporins and Penicillin) it can be inferred that the haloarchaeon develops multi drug resistance on exposure to simulated microgravity. Exposure to simulated microgravity thus increases the antibiotic resistance and renders the culture as Multi-Drug Resistant (MDR) strain of *H. argentinensis* RR10.

Microbial mechanisms of resistance to antibiotics are plasmid encoded resistance, cell wall alterations, and changes in permeability of membranes, production of antibiotic inactivating enzymes and activation of drug efflux pumps (Mapara et al. 2015). Multidrug resistance in microorganisms has garnered impending importance in public health during recent times due to its inherent role in causing life threatening diseases and outbreaks. The antibiotic resistance of haloarchaea is thus of paramount significance as its role in pathogenesis and etiology in human disease is still largely unknown. Besides, considering that these organisms are used to study astrobiology and as exposure to microgravity increases their pathogenicity and antimicrobial resistance (Wilson et al. 2007) further studies related to the mechanisms of drug resistance in these haloarchaea is imperative. Studies related to the presence of antibiotic efflux pumps in haloarchaea are meagre. Antibiotic efflux pump has been detected only in *Haloferax volcanii* that mediates the transport of doxorubicin, vinblastin, vincristin, ethidium and monensin **(**Miyauchi et al. 1992). In the present study, we have reported the presence of antibiotic efflux pump in *H. argentinensis* RR10 using the modified ethidium bromide cartwheel assay for the first time. The increase in pigmentation due to synthesis of carotenoid was also notable in haloarchaea exposed to SMG. These carotenoid pigments are involved in cellular protection of the haloarchaea against oxidative damage caused by deleterious effects of stress.

## Conclusion

The present study is presumably the first report on studying the growth kinetics and physiological response of *H. argentinensis* RR10 in simulated microgravity in a clinostat. It is difficult to explain how the mechanical forces of fluid shear transmits intracellular signals to microbial cells at molecular level. The results of the present study indicate that when *H. argentinensis* RR10 is exposed to simulated microgravity, it predominantly responds by acceleration of growth, increase in cellular pigmentation, activation of ‘salt-in’ strategy of osmoadaptation and upregulation of transcription related ribosomal proteins. Simulated microgravity also causes the increase in antibiotic resistance of the organism and upregulation of antibiotic efflux pumps. The results suggest that there may be a possibility of cross talk between microbial signal transduction systems and future studies might reveal common mechanotransduction themes between these systems and those used to sense and respond to low-shear stress and changes in gravitation forces (Nickerson et al. 2004).
